# CO_2_-Fixation Strategies in Energy Extremophiles: What Can We Learn From Acetogens?

**DOI:** 10.3389/fmicb.2020.00486

**Published:** 2020-04-03

**Authors:** Olivier N. Lemaire, Marion Jespersen, Tristan Wagner

**Affiliations:** Microbial Metabolism Group, Max Planck Institute for Marine Microbiology, Bremen, Germany

**Keywords:** acetogenic bacteria, hydrogenotrophic methanogens, CO_2_-fixation, formate dehydrogenase, evolution, biotechnology, coupled reaction

## Abstract

Domestication of CO_2_-fixation became a worldwide priority enhanced by the will to convert this greenhouse gas into fuels and valuable chemicals. Because of its high stability, CO_2_-activation/fixation represents a true challenge for chemists. Autotrophic microbial communities, however, perform these reactions under standard temperature and pressure. Recent discoveries shine light on autotrophic acetogenic bacteria and hydrogenotrophic methanogens, as these anaerobes use a particularly efficient CO_2_-capture system to fulfill their carbon and energy needs. While other autotrophs assimilate CO_2_ via carboxylation followed by a reduction, acetogens and methanogens do the opposite. They first generate formate and CO by CO_2_-reduction, which are subsequently fixed to funnel the carbon toward their central metabolism. Yet their CO_2_-reduction pathways, with acetate or methane as end-products, constrain them to thrive at the “thermodynamic limits of Life”. Despite this energy restriction acetogens and methanogens are growing at unexpected fast rates. To overcome the thermodynamic barrier of CO_2_-reduction they apply different ingenious chemical tricks such as the use of flavin-based electron-bifurcation or coupled reactions. This mini-review summarizes the current knowledge gathered on the CO_2_-fixation strategies among acetogens. While extensive biochemical characterization of the acetogenic formate-generating machineries has been done, there is no structural data available. Based on their shared mechanistic similarities, we apply the structural information obtained from hydrogenotrophic methanogens to highlight common features, as well as the specific differences of their CO_2_-fixation systems. We discuss the consequences of their CO_2_-reduction strategies on the evolution of Life, their wide distribution and their impact in biotechnological applications.

## Introduction

CO_2_, the most oxidized state of carbon, has become a major concern to society due to its greenhouse gas properties and its increasing accumulation in our atmosphere since the 20th-century. Efficient CO_2_-sequestration techniques, as well as concomitant applications in biochemical synthesis and alternative energy source storage, being developed to reduce its impact on global warming (Schuchmann and Müller, 2013). Yet CO_2_ is a stable, inert molecule. The few applicable chemical processes allowing its unfavorable fixation (like the Monsanto and Cativa processes) require high temperatures and pressures as well as expensive and polluting catalysts while only exhibiting moderate catalytic rates (Appel et al., 2013; Fujita et al., 2013; Schuchmann and Müller, 2013). New alternative chemistry based on metal-organic framework (Hou et al., 2019) or transition metal-free catalysis (Cherubini-Celli et al., 2018) are upcoming and might be applied in the near future. Nevertheless, none of these artificial processes matches the efficiency of their biological counterpart.

At least six different autotrophic carbon fixation pathways exist among the domains of Life (Berg et al., 2010; Fuchs, 2011; Appel et al., 2013). The most common scenario is a two-step process where CO_2_ is branched on a reactive group (carboxylation) and then reduced (e.g., Calvin–Benson–Bassham or the 3-hydroxypropionate 4-hydroxybutyrate cycle). To date, there is only one exception that uses the reverse way, CO_2_-reduction before carboxylation: the reductive acetyl-CoA pathway. This pathway constitutes the “cheapest” option to fix CO_2_ in term of energy-consumption and is thought to be the most ancient one (Berg et al., 2010; Martin and Thauer, 2017).

**FIGURE 1:**
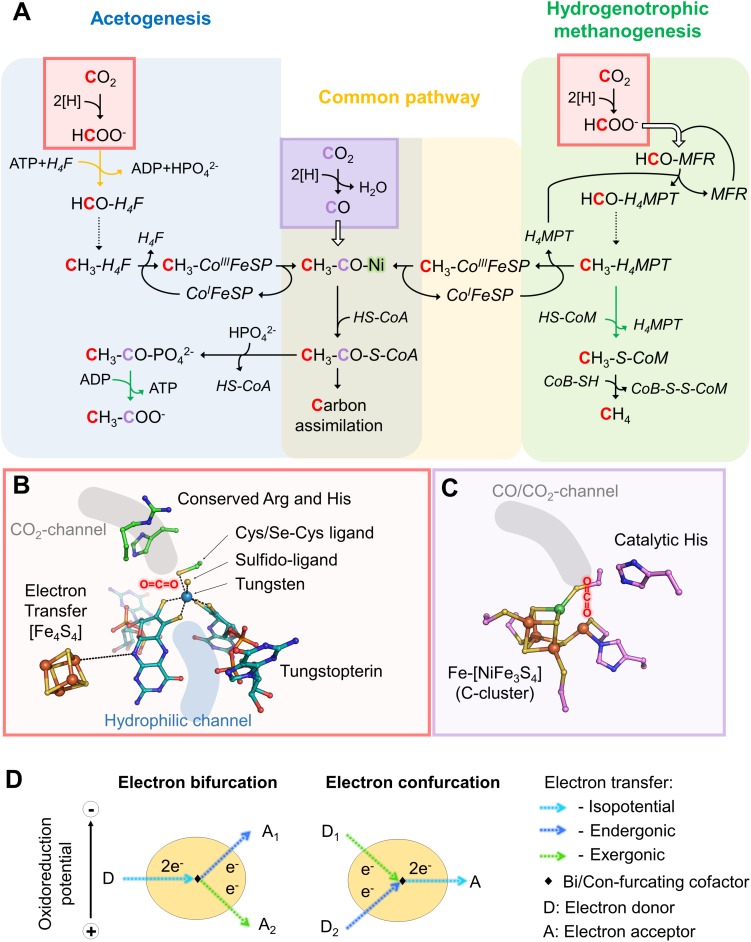
Variations in the reductive acetyl-CoA pathway between acetogenic bacteria and hydrogenotrophic methanogens, implicated active sites and mechanisms. **(A)** Differences in the reductive acetyl-CoA pathway between acetogenic bacteria (left and middle) and methanogenic archaea (right and middle). Acetogens and methanogens share a conserved “carbonyl” branch (common pathway) used to build biomass for both and to conserve energy for acetogens. The green arrows correspond to reactions coupled to energy-conservation (ATP or electrochemical ion gradient generation across the membrane) and the orange one to ATP hydrolysis-coupled reaction. Dashed arrows correspond to three successive reactions: dehydration and two reduction steps. White arrows indicate the usage of an internal channeling system between two active sites. Red and purple squares highlight CO_2_-reduction events, in red Fdh reaction and in purple the CODH reaction. The ACS contains the A-cluster harboring the binuclear Nickel center highlighted by a green glow. The cofactors involved in these processes are: tetrahydrofolate (H_4_F), tetrahydromethanopterin (H_4_MPT), coenzyme A (CoA-SH), methanofuran (MFR), reduced/oxidized corrinoid FeS containing protein (Co^I^/CH_3_-Co^III^-FeSP), coenzyme B (CoB-SH), coenzyme M (CoM-SH). **(B)** Close up of a Fdh catalytic site (PDB code 5T5I) containing the tungstopterin, which could be replaced by molybdopterin for other Fdh. Carbons are colored in green for the residues involved in the catalysis and dark cyan for the tungstopterin. Dashed line between the [Fe_4_S_4_]-cluster and the pterin represents the hypothetic electron transfer from the cluster to the tungstopterin. **(C)** Close up of the catalytic site of CODH from *Moorella thermoacetica* (PDB code 1MJG) containing the C-cluster. Carbons from protein residues are colored in light pink. For both panels, **B** and **C**, nitrogen, oxygen, phosphorous, sulfur, iron, tungsten, and nickel are colored as dark blue, red, light orange, yellow, orange, metallic blue, and green, respectively. A molecule highlights the putative CO_2_ position in both panels. **(D)** Scheme of electron bifurcation/confurcation mechanism. During electron bifurcation, a two-electron transfer from an electron donor (D) is bifurcated by a specific cofactor to both endergonic and exergonic one-electron transfers to two different acceptors (A_1_ and A_2_). The overall reaction is slightly exergonic. The opposite reaction occurs during electron confurcation.

The reductive acetyl-CoA pathway has two CO_2_ entry points: the methyl-branch, where a reductive cascade turns CO_2_ in a methyl-group (Figure 1A, reaction from the CO_2_-activation to the methyl-H_4_F for acetogens or methyl-H_4_MPT for methanogens) and the carbonyl-branch. In the latter, CO_2_ is converted into carbon monoxide (CO; Figure 1A), further combined with the methyl group and Coenzyme A (CoA) to ultimately produce acetyl-CoA, the “turntable” of the central carbon metabolism (Ragsdale and Pierce, 2008; Thauer et al., 2008; Berg et al., 2010; Sousa et al., 2013; Fuchs and Berg, 2014).

Hydrogenotrophic methanogens (Euryarchaea, simplified as methanogens below) and autotrophic acetogens (Bacteria, simplified as acetogens below) use the reductive acetyl-CoA pathway to derive their cellular carbon and energy by growing on H_2_ plus CO_2_. The final product for methanogens and acetogens are methane and acetate, respectively. Under physiological conditions, such metabolism provides less than half a molecule of ATP per acetate/methane, constraining these organisms to live at the “thermodynamic limits of Life” (Buckel and Thauer, 2013; Schuchmann and Müller, 2014). Nevertheless, methanogens and acetogens are found in various ecological niches, ranging from rumen to deep-sea volcanoes and they are crucial actors in organic matter conversion and element cycling (e.g., carbon assimilation and nitrogen fixation). Despite drastically low energy yields, their doubling time is surprisingly short: ranging from only one to a few hours under laboratory conditions (Thauer et al., 2008; Basen et al., 2018).

The energy metabolism of acetogens and methanogens was puzzling for a long time until the discovery of energy conserving enzymes (i.e., Rnf and Ech membrane complexes), which use low-potential electrons from ferredoxins, reduced by H_2_ oxidation via flavin-based electron bifurcation (Figure 1D). The use of low-potential electrons provided a rational explanation as to how these organisms derive enough energy to survive and grow under such stringent metabolic conditions (Buckel and Thauer, 2013, 2018; Schuchmann and Müller, 2014; Peters et al., 2018). Considered to be among the first metabolic processes, methanogenesis and acetogenesis might have been crucial for shaping ecosystems since the first Lifeforms arose.

This review summarizes our current understanding of the CO_2_-activation steps orchestrated by these fantastic machineries, which evolved to fulfill the physiological needs for carbon-assimilation and energy-conservation. The structural knowledge gathered from hydrogenotrophic methanogens provides insights in the shared and distinct features between the acetogenic and methanogenic CO_2_-conversion systems, due to both metabolic adaptation and ecological specialization.

## The Co_2_-Reduction/Fixation Complex in Methanogens

**FIGURE 2:**
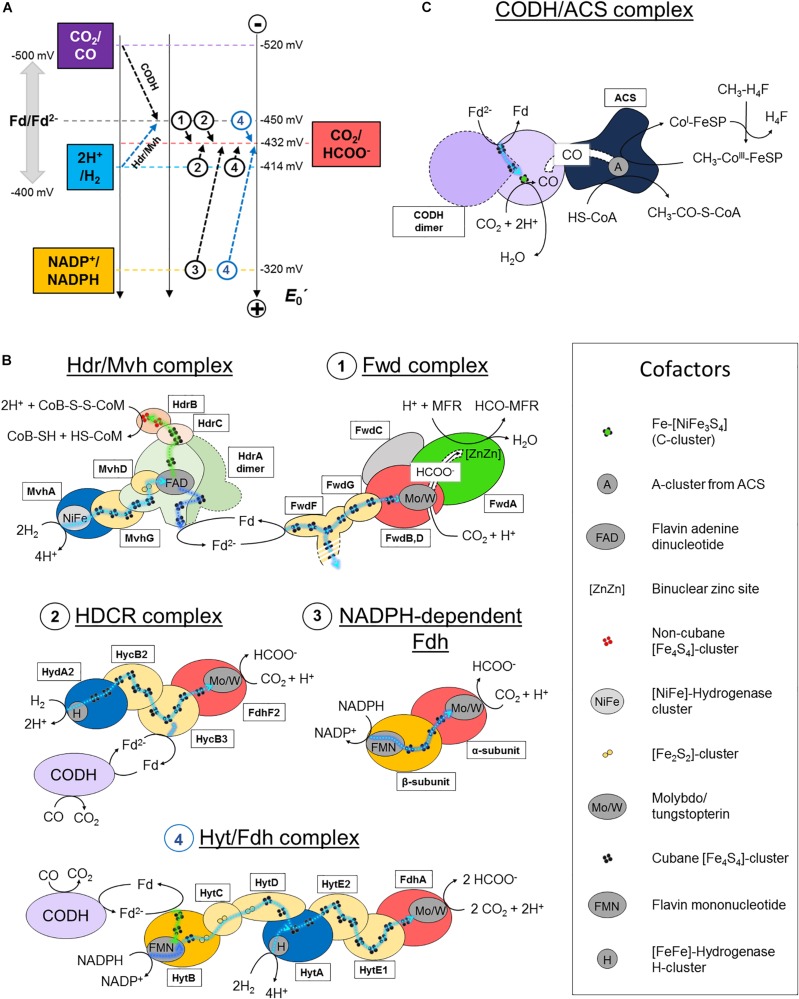
CO_2_-activation strategies in hydrogenotrophic methanogens and acetogens. **(A)** Standard redox potential (*E*_0_′) of redox couples implicated in CO_2_-activation in methanogenic and acetogenic processes. Dashed arrows schematize the reactions performed by the enzymes listed in panel **B**. A name or circled number indicates which complex is implicated. The blue arrows correspond to a coupled electron confurcating reaction. To simplify the scheme, the endergonic reaction of the Hdr/Mvh complex (CoB-S-S-CoM/CoB-SH + CoM-SH = −140 mV) has been omitted. Standard redox potentials were taken from Schuchmann and Müller (2014). Ferredoxin can exhibit potentials ranging from −400 to −500 mV, depending on the organism. An averaged potential of −450 mV is thus used in the figure. **(B,C)** Schemes of the characterized and putative organizations of the enzymes involved in CO_2_-reduction in hydrogenotrophic methanogens and acetogens. Catalytic subunits are colored according to their substrate as in panel **A**. All electron transfers except for con/bifurcation events are shown as cyan dashed lines. For con/bifurcation events, the endergonic reactions are colored in dark blue and exergonic in light green, as illustrated in Figure 1D. Due to the absence of acetogenic structural data, hypothetic architectures are represented based on their original publications, biochemical data and homologies (Yamamoto et al., 1983; Schuchmann and Müller, 2013; Wang et al., 2013). Monomeric forms are schematized. The localization of the electron confurcation event in the Hyt complex is purely hypothetic. The [NiFe]-hydrogenase module from the Hdr/Mvh complex can be replaced by a Fdh. All known cofactors involved in the different reactions of panels **B** and **C** are listed. Fd stands for oxidized ferredoxin and Fd^2−^ for reduced ferredoxin.

The entire energy metabolism of methanogens relies on highly efficient CO_2_-capture. This challenging task is overcome by the formyl-methanofuran dehydrogenase (Fwd) complex catalyzing both the reduction of CO_2_ and the conversion of formate (HCOO^–^) into a formyl group (Figures 1A, 2). So far, two isoforms of this enzyme are described, containing either a molybdenum- or tungsten-dependent formate dehydrogenase (Fdh) subunit (Bertram et al., 1994; Thauer et al., 2008; Leimkühler and Iobbi-Nivol, 2016; Wagner et al., 2018). Depending on the organism, the molybdo/tungstopterin cofactor can be coordinated by a cysteine or seleno-cysteine. Unlike nearly all described Fdh that perform formate oxidation releasing CO_2_, methanogenic and acetogenic Fdh physiologically run toward CO_2_-fixation. Until now, only a few enzymes found in the *Synthrophobacter* genus share this feature (de Bok et al., 2003).

The reaction remained a mystery for a long time: how can the enzyme couple formate to the C1-carrier methanofuran (MFR) without any ATP investment (Bertram et al., 1994)? The secret was eventually unraveled by its crystal structure (Wagner et al., 2016). The overall complex is constituted of an unprecedented electron transfer apparatus containing a total of 46-[Fe_4_S_4_] clusters flanked by two catalytic modules, a tungstopterin-dependent Fdh and a binuclear metallo-hydrolase. Based on the molecular details, a scenario of the reaction has been proposed where; (1) CO_2_ is funneled to the active site of Fdh by a selective, hydrophobic channel; (2) CO_2_ is reduced to formate (Figures 1A,B, 2A,B; Wagner et al., 2016); (3) a second hydrophilic tunnel channels and accumulates formate at the active site of the metallo-hydrolase; and (4) formate is condensed on the amino-group of MFR as a formyl-group. The accumulation of formate is predicted to thrive the conversion of formate to a formyl group on MFR without the investment of ATP, an endergonic reaction under standard conditions. However, since Fdh are reversible, the driver of the overall reaction is the electron donor.

From MFR the formyl group is transferred to the second C1-carrier tetrahydromethanopterin (H_4_MPT), successively dehydrated and fully reduced (i.e., by the F_420_ cofactor or alternatively with H_2_ by the [Fe]-hydrogenase) to a methyl group (dashed line, Figure 1A). The methyl-H_4_MPT represents the crossroad between carbon-assimilation and energy conservation (Thauer, 2012). The latter is formed during the methyl group transfer from nitrogen-bound methyl-H_4_MPT to the thiol group of the coenzyme-M acceptor. The methyl-transfer is coupled to a sodium translocation across the membrane, used to feed the ATP-synthase, which is generating only half an ATP per processed C1-unit (Schäfer et al., 1999). Finally, methylated coenzyme-M becomes oxidized to a heterodisulfide with coenzyme-B (CoB-S-S-CoM), releasing methane by using the F_430_-cofactor (Thauer et al., 2008; Thauer, 2012; Sousa et al., 2013).

## Electron-Bifurcation Fuels Methanogenic Co_2_ Fixation

The electron donor for the Fwd complex is predicted to be reduced ferredoxin or a direct electron transfer by the heterodisulfide reductase (Costa et al., 2010; Kaster et al., 2011; Milton et al., 2018). Two versions of this enzyme have been described in hydrogenotrophic methanogens depending on the coupled electron donor: a [NiFe]-hydrogenase or a Fdh (the structurally characterized hydrogenase-dependent one called the Hdr/Mvh complex is shown in Figures 1, 2B).

The overall process starts with the transfer of two electrons from the donor (H_2_ or formate) to a flavin. Flavin-based electron bifurcation then splits the two electrons at different potentials (Figures 1D, 2B). The high-potential electron is used for the exergonic reduction of the heterodisulfide. The low-potential electron reduces ferredoxin or might even be directly delivered to the 46-[Fe_4_S_4_] relay of the formyl-methanofuran dehydrogenase to allow CO_2_-capture. The whole reaction is performed in two rounds (Wagner et al., 2017).

## Diversity of Co_2_-Activation Systems in Acetogens

Like methanogens, acetogenic bacteria perform initial CO_2_-reduction via molybdo/tungstopterin-dependent Fdh. However, in contrast to methanogens, all described acetogenic CO_2_-reducing systems produce detectable formate, indicating that CO_2_-reduction and formate condensation are uncoupled (Yamamoto et al., 1983; Schuchmann and Müller, 2013, 2014; Wang et al., 2013). Formate conversion into a formyl group on a C1-carrier (i.e., tetrahydrofolate, H_4_F) is thermodynamically unfavorable and acetogens must therefore invest one ATP. Then, the successive dehydration and reduction into methenyl, methylene and finally methyl group can occur, similar to the methanogenic process but involving different systems, reductants and cofactors (Mock et al., 2014; Schuchmann and Müller, 2014). The methyl group is further fused to CO and CoA by the CO dehydrogenase/acetyl-CoA synthase complex (CODH/ACS) to form acetyl-CoA (see below, Figures 1, 2C; Ragsdale, 2008; Can et al., 2014).

Despite a common Fdh module, acetogens evolved their CO_2_-fixation system in many variations, a real example of “mix and match” from the redox module toolbox, as already shown for sulfate-reducing organisms (Grein et al., 2013). Some of them have been reported to be molybdopterin dependent (e.g., *Acetobacterium woodii*) while others require tungstopterin (e.g., *Moorella thermoacetica*, *Clostridium autoethanogenum*, etc.). There are selenium-dependent or selenium-free formate dehydrogenases and some organisms encode both (Yamamoto et al., 1983; Schuchmann and Müller, 2013; Wang et al., 2013). This diversity allows the use of different electron donors (Schuchmann and Müller, 2014).

*Acetobacterium woodii* uses a hydrogen-dependent CO_2_-reductase (HDCR) which couples the oxidation of H_2_ to the reduction of CO_2_ (Schuchmann and Müller, 2013). The enzyme is composed of a selenocysteine–molybdopterin dependent Fdh linked to a [FeFe]-hydrogenase via an electron bridge (Figure 2B, 2). The genome also encodes a cysteine-dependent Fdh isoform, supposedly expressed under selenium deprivation. The reduction of CO_2_ to formate with H_2_ is slightly endergonic at standard conditions (Figure 2A). However, at the relatively important threshold concentration of H_2_, necessary for acetogenesis in *A. woodii* (measured at 0.0025 Bar, around thirty times superior to the threshold of hydrogenotrophic methanogens; Thauer et al., 2008), the equilibrium concentration of generated formate becomes sufficient to fuel the methyl branch of the pathway (Schuchmann and Müller, 2014). The enzyme can also perform carbon fixation by ferredoxin oxidation, albeit exhibiting a 1000-times lower reaction rate (Figure 2B, 2). This ability is thought to be crucial in presence of CO, a strong inhibitor of hydrogenases. The coupling of HDCR with CODH is an efficient way to regenerate reduced ferredoxin.

*Moorella thermoacetica* contains a two subunit NADPH-dependent Fdh containing selenocysteine and tungstopterin cofactor, which catalyzes the reversible formate generation through NADPH oxidation (Figure 2B, 3; Thauer, 1972; Yamamoto et al., 1983). Despite being thermodynamically highly unfavorable under standard conditions, it appears that the NADPH-dependent Fdh is the only formate generating enzyme in *M. thermoacetica*. High NADPH/NADP^+^ and CO_2_/formate ratios are necessary to push the reaction toward carbon reduction.

*Clostridium autoethanogenum* exploits a seven subunit complex (Hyt/Fdh), the so far most complicated formate-generating system in acetogens (Figure 2B, 4; Wang et al., 2013; Schuchmann and Müller, 2014). The selenium-dependent tungstopterin-containing Fdh module performs CO_2_-reduction by receiving electrons from H_2_-oxidation via a [FeFe]-hydrogenase subunit (similar to HDCR) or by concomitant oxidation of NADPH and ferredoxin through an internal confurcation event (Figures 1D, 2B, 4). Like in HDCR, the reduced ferredoxin could directly come from CO-oxidation by the CODH. Where and how the electron confurcation is carried out is still unresolved, as the only known flavin cofactor present in the complex (in HytB) is thought to be not involved (Wang et al., 2013). A novel type of electron bifurcation is thus suspected, one that is similar to the related electron-bifurcating hydrogenase. The structural features of this bifurcation mechanism have to be deciphered and as said by Buckel and Thauer (2018): “A crystal structure is urgently needed to solve this problem.”

## A Common Co_2_-Fixation System: The Co-Dehydrogenase/Acetyl-Coa Synthase

While methanogens and acetogens employ different strategies to reduce CO_2_ for the methyl-branch, the activation step of the carbonyl-branch, catalyzed by Ni,Fe-containing CODH, is remarkably conserved (Lindahl, 2002; Jeoung et al., 2019).

The initial CO_2_-reduction to CO, powered by low-potential electrons from ferredoxins or flavodoxins (Ragsdale et al., 1983; Can et al., 2014; Schuchmann and Müller, 2014), occurs at the C-cluster composed of a Fe-[NiFe_3_S_4_] (Figure 1C). CO is transferred to the ACS by a long internal hydrophobic channel (Figure 2C; Doukov et al., 2002; Can et al., 2014). Here, it is fixed on the A-cluster, which is composed of a Ni-[Fe_4_S_4_] cluster bridged to another Ni atom (Ragsdale and Pierce, 2008). Ultimately, the ACS forms acetyl-CoA by associating the CO-ligand, CoA and the methyl-ligand from the methyl-branch. A cobalamin-containing FeS protein (CoFeSP) serves as a shuttle for the methyl group between the Methyl-H_4_F and the ACS. The transfer mechanism from the CoFeSP to the ACS is so far unknown. The enzyme thus performs the biological equivalent of the Monsanto and Cativa processes, where CO and methanol are converted to acetate by metal-based catalysts (Appel et al., 2013).

Even if the overall reaction is the same between methanogens and acetogens some subtleties concerning the CODH/ACS composition exist. According to the classification of Lindahl (2002), archaea and predominantly methanogens use preferentially Ni,Fe-CODH of Class I and II (also called acetyl-CoA decarbonylases/synthases), which consist of five different subunits that form oligomeric complexes of approximately 2-MDa. This super-complex contains the CODH/ACS (Doukov et al., 2002; Can et al., 2014), the CoFeSP and the enzyme responsible for methyl-transfer from methyl-H_4_F to cobalamin. These three sub-complexes are separated in acetogenic systems. The CODH subunit in methanogens contains two extra [Fe_4_S_4_]-clusters, putatively implicated in the rerouting of electrons. Acetogenic complexes have been extensively studied (Ragsdale and Kumar, 1996; Ragsdale and Pierce, 2008) thanks to a few available crystal structures of the whole CODH/ACS complex from *M. thermoacetica* (Doukov et al., 2002; Darnault et al., 2003; Kung et al., 2009) and the knowledge gathered on this enzyme has already been reviewed (Can et al., 2014).

Beside these slight differences, all classes of the CODH/ACS complex are thought to be homologous and thus may have been acquired from a common ancestor (Sousa et al., 2013).

## Conclusion and Perspectives

As previously depicted, the Fdh subunit and CODH/ACS complex are conserved in methanogens and acetogens. These elementary modules are therefore thought to have evolved before the divergence of acetogens and methanogens, thus in the Last Universal Common Ancestor (LUCA) (Sousa et al., 2013; Martin and Thauer, 2017). Because they harbor “ancestral” cofactors like Fe-S clusters or tungstopterin and since the substrates H_2_ and CO_2_ should have been abundant in Early Earth, these pathways are considered to be among the first, if not the first, biological energetic processes (Fuchs, 2011; Sousa et al., 2013; Martin and Thauer, 2017). Understanding the mechanisms and limitations of methanogenesis and acetogenesis will help to unravel the fundamental questions of how Life arose from the pre-existing inorganic world and could provide information about its first evolutionary steps in the new organic one.

While the carbonyl-branch of the reductive acetyl-CoA pathway might be an early and highly conserved invention in LUCA, the methyl-branch is not. Here, methanogens and acetogens use non-homologous enzymes to perform similar reactions. This parallel evolution gave birth to a variety of formate-generating, CO_2_-reducing enzymes, albeit using similar modules (Sousa et al., 2013) and invented different strategies for C1-reduction and formate condensation by the use of different C1-carriers. The evolutionary plasticity of the methyl-branch compared to the strict conservation of the carbonyl-branch might derive from its requirement for low-potential electrons. Because of the low-potential of the CO_2_/CO couple, the CODH could not adapt to a partner other than ferredoxin for CO generation, while the CO_2_-reduction to formate can accommodate different electron donors, allowing variability of enzymes according to the metabolic needs for physiological requirements.

Furthermore, the functional modules coupled to Fdh systems might be the foundation for other “modern” enzymes, from the formate-hydrogen lyase complex to the respiratory complex I (Marreiros et al., 2016). Elucidating methanogenic and acetogenic enzymes has therefore the potential to provide hints to how the ancestral energetic pathways diversified, thereby creating new processes and gradually giving birth to the plethora of bio-energetically important complexes.

A striking difference between formate generating enzymes from acetogens and methanogens is the energy investment. While methanogens bypass the latter (Figure 1A), acetogens need to sacrifice one ATP to allow formate fixation. They counterbalance this energy loss via substrate level phosphorylation of acetyl-phosphate in the last step of acetogenesis. In comparison, no ATP is generated through methanogenesis (Figure 1A). Nevertheless, ATP sparing is critical for energy-limited extremophiles and one could ask why acetogens did not develop an equivalent of the Hdr/Mvh/Fwd coupling system. The explanation could come from the use of low-potential ferredoxins. The last step of methanogenesis releases CoB-S-S-CoM, which is recycled by the heterodisulfide reductase (downhill reaction) with the concomitant generation of low-potential electrons (uphill reaction). Most of these low-potential electrons generated in the cell are assumed to be dedicated for the CO_2_-fixation (Figure 2B, 1; Thauer, 2012).

Acetogenic bacteria are restricted to ecological niches with higher H_2_ pressure than methanogens. The main reason is that electron bifurcating [FeFe]-hydrogenases are necessary for ferredoxin reduction (Schuchmann and Müller, 2012, 2014), the electron acceptor for the downhill reaction being NAD(P)^+^. According to the current knowledge, the uphill electron generated during the flavin-based electron bifurcation could have a lower potential if the electron downhill is the heterodisulfide (*E*_0_′ ≈ −140 mV) compared to NAD(P)^+^ (*E*_0_′ = −320 mV). Therefore, the ferredoxins reduced via electron bifurcation in methanogens are expected to have higher reducing power compared to acetogens. Thus, in the latter the potential could not be low enough to allow both, formate generation and conversion to formyl group, unlike in methanogens. Thus, despite sparing one ATP, coupling formate generation and fixation may be not favorable for acetogenic bacteria and will not sustain a metabolic high-flow toward acetyl-CoA synthesis.

A way to bypass H_2_ is CO-oxidation. To handle CO, acetogens use different strategies. *A. woodii* thrives episodically on weak CO concentrations, possible due to the reversibility of the CO inhibition of the HDCR system and the ability to oxidize ferredoxin, albeit with a weak turnover (Figure 2B, 2; Schuchmann and Müller, 2013; Bertsch and Müller, 2015; Ceccaldi et al., 2017). Acetogenic bacteria, which use CO as substrate, like *M. thermoacetica* and *C. autoethanogenum*, exhibit metabolic adaptations. For instance, albeit it has not been tested so far, the NADPH-dependent Fdh system from *M. thermoacetica* should be insensitive to CO as it is not directly using the CO-sensitive hydrogenase, like the HDCR. However, the enzyme depends on a high NADPH/NADP^+^ ratio or high pressure of CO_2_. The Hyt/Fdh system from *C. autoethanogenum*, a chimera between HDCR and the electron bifurcating/confurcating hydrogenase (Figure 2B, 4), shows a reactional plasticity by switching from the CO-sensitive hydrogenase to NADPH plus ferredoxin oxidation to drive CO_2_-reduction despite inhibition (Wang et al., 2013). Interestingly, to date, a formyl group generation directly driven by CO-oxidation has never been found in any CO fermenting acetogen. Still, the low redox potential of the CO_2_/CO couple could allow an Fwd-like coupled mechanism, sparing a molecule of ATP, crucial for such energetic extremophiles.

The diversity of the electron-donating Fdh systems reflects and allowed the widespread distribution of these microbes, from H_2_ rich to CO saturated niches. However, their dependence on oxygen-sensitive cofactors constrains them to strictly anaerobic but also metal-rich environments, since such carbon fixation pathways require more metallic cofactors than the others. Studying the diversity of these systems provides modern snapshots of the evolution of such “ancestral” organisms to accommodate various ecological niches.

Because syngas (H_2_/CO_2_/CO) is the main source of carbon and energy for hydrogenotrophic methanogens and acetogens, they are excellent “bio-converters.” For instance, acetogens turn industrial waste gases, rich in H_2_, CO and CO_2_, to butanediol, ethanol or acetate, potential biofuels or starting points for new chemical synthesis (Wang et al., 2013; Mock et al., 2015; Liew et al., 2016). With the discovery of genetically tractable acetogens (Liew et al., 2016; Basen et al., 2018) the possibilities for bio-compound synthesis, and bioremediation are expanding.

Moreover, acetogenic CO_2_-activation systems as HDCR and Hyt/Fdh are a treasure trove to realize the Holy Grail reaction of our century: the reversible hydrogenation of CO_2_ to formate, offering a stable way to store energy with the concomitant advantage of trapping the greenhouse gas (Schuchmann and Müller, 2013; Müller, 2019).

Studies of acetogenic physiology and carbon fixation pathways are still an ongoing growing field. More work has to be conducted to truly understand their enzymes, metabolic fluxes, the molecular juggling of their reactions and their limitations. It is crucial to ensure the success of biotechnological applications, including synthetic biology, that will – let’s hope – bring a brighter future.

## Author Contributions

All authors participated to the manuscript writing.

## Conflict of Interest

The authors declare that the research was conducted in the absence of any commercial or financial relationships that could be construed as a potential conflict of interest.

## References

[B1] Appel A. M. Bercaw J. E. Bocarsly A. B. Dobbek H. DuBois D. L. Dupuis M. (2013). Frontiers, opportunities, and challenges in biochemical and chemical catalysis of CO2 fixation. *Chem. Rev.* 113 6621–6658. 10.1021/cr300463y 23767781 PMC3895110

[B2] Basen M. Geiger I. Henke L. Müller V. (2018). A genetic system for the thermophilic acetogenic bacterium *Thermoanaerobacter kivui*. *Appl. Environ. Microbiol.* 84 e2210–e2217. 10.1128/AEM.02210-17 29150512 PMC5772241

[B3] Berg I. A. Kockelkorn D. Ramos-Vera W. H. Say R. F. Zarzycki J. Hügler M. (2010). Autotrophic carbon fixation in archaea. *Nat. Rev. Microbiol.* 8 447–460. 10.1038/nrmicro2365 20453874

[B4] Bertram P. A. Karrasch M. Schmitz R. A. Böcher R. Albracht S. P. Thauer R. K. (1994). Formylmethanofuran dehydrogenases from methanogenic archaea. Substrate specificity, EPR properties and reversible inactivation by cyanide of the molybdenum or tungsten iron-sulfur proteins. *Eur. J. Biochem.* 220 477–484. 10.1111/j.1432-1033.1994.tb18646.x 8125106

[B5] Bertsch J. Müller V. (2015). CO metabolism in the acetogen *Acetobacterium woodii*. *Appl. Environ. Microbiol.* 81 5949–5956. 10.1128/AEM.01772-15 26092462 PMC4551271

[B6] Buckel W. Thauer R. K. (2013). Energy conservation via electron bifurcating ferredoxin reduction and proton/Na+ translocating ferredoxin oxidation. *Biochim. Biophys. Acta* 1827 94–113. 10.1016/j.bbabio.2012.07.002 22800682

[B7] Buckel W. Thauer R. K. (2018). Flavin-based electron bifurcation, a new mechanism of biological energy coupling. *Chem. Rev.* 118 3862–3886. 10.1021/acs.chemrev.7b00707 29561602

[B8] Can M. Armstrong F. A. Ragsdale S. W. (2014). Structure, function, and mechanism of the nickel metalloenzymes, CO dehydrogenase, and acetyl-CoA synthase. *Chem. Rev.* 114 4149–4174. 10.1021/cr400461p 24521136 PMC4002135

[B9] Ceccaldi P. Schuchmann K. Müller V. Elliott S. J. (2017). The hydrogen dependent CO2 reductase: the first completely CO tolerant FeFe-hydrogenase. *Energy Environ. Sci.* 10 503–508. 10.1039/C6EE02494G

[B10] Cherubini-Celli A. Mateos J. Bonchio M. Dell’Amico L. Companyó X. (2018). Transition metal-free CO2 fixation into new carbon-carbon bonds. *ChemSusChem* 11 3056–3070. 10.1002/cssc.201801063 29882632

[B11] Costa K. C. Wong P. M. Wang T. Lie T. J. Dodsworth J. A. Swanson I. (2010). Protein complexing in a methanogen suggests electron bifurcation and electron delivery from formate to heterodisulfide reductase. *Proc. Natl. Acad. Sci. U.S.A.* 107 11050–11055. 10.1073/pnas.1003653107 20534465 PMC2890747

[B12] Darnault C. Volbeda A. Kim E. J. Legrand P. Vernède X. Lindahl P. A. (2003). Ni-Zn-[Fe4-S4] and Ni-Ni-[Fe4-S4] clusters in closed and open α subunits of acetyl-CoA synthase/carbon monoxide dehydrogenase. *Nat. Struct. Biol.* 10 271–279. 10.1038/nsb912 12627225

[B13] de Bok F. A. Hagedoorn P. L. Silva P. J. Hagen W. R. Schiltz E. Fritsche K. (2003). Two W-containing formate dehydrogenases (CO2-reductases) involved in syntrophic propionate oxidation by *Syntrophobacter fumaroxidans*. *Eur. J. Biochem.* 270 2476–2485. 10.1046/j.1432-1033.2003.03619.x 12755703

[B14] Doukov T. I. Iverson T. M. Seravalli J. Ragsdale S. W. Drennan C. L. (2002). A Ni-Fe-Cu center in a bifunctional carbon monoxide dehydrogenase/acetyl-CoA synthase. *Science* 298 567–572. 10.1126/science.1075843 12386327

[B15] Fuchs G. (2011). Alternative pathways of carbon dioxide fixation: insights into the early evolution of life? *Annu. Rev. Microbiol.* 65 631–658. 10.1146/annurev-micro-090110-102801 21740227

[B16] Fuchs G. Berg I. A. (2014). Unfamiliar metabolic links in the central carbon metabolism. *J. Biotechnol.* 192(Pt B), 314–322. 10.1016/j.jbiotec.2014.02.015 24576434

[B17] Fujita E. Muckerman J. T. Himeda Y. (2013). Interconversion of CO2 and formic acid by bio-inspired Ir complexes with pendent bases. *Biochim. Biophys. Acta* 1827 1031–1038. 10.1016/j.bbabio.2012.11.004 23174332

[B18] Grein F. Ramos A. R. Venceslau S. S. Pereira I. A. (2013). Unifying concepts in anaerobic respiration: insights from dissimilatory sulfur metabolism. *Biochim. Biophys. Acta* 1827 145–160. 10.1016/j.bbabio.2012.09.001 22982583

[B19] Hou S. L. Dong J. Zhao B. (2019). Formation of C-X bonds in CO2 chemical fixation catalyzed by metal-organic frameworks. *Adv. Mater.* 32:e1806163. 10.1002/adma.201806163 31216093

[B20] Jeoung J. H. Martins B. M. Dobbek H. (2019). Carbon monoxide dehydrogenases. *Methods Mol. Biol.* 1876 37–54. 10.1007/978-1-4939-8864-8_3 30317473

[B21] Kaster A. K. Moll J. Parey K. Thauer R. K. (2011). Coupling of ferredoxin and heterodisulfide reduction via electron bifurcation in hydrogenotrophic methanogenic archaea. *Proc. Natl. Acad. Sci. U.S.A.* 108 2981–2986. 10.1073/pnas.1016761108 21262829 PMC3041090

[B22] Kung Y. Doukov T. I. Seravalli J. Ragsdale S. W. Drennan C. L. (2009). Crystallographic snapshots of cyanide- and water-bound C-clusters from bifunctional carbon monoxide dehydrogenase/acetyl-CoA synthase. *Biochemistry* 48 7432–7440. 10.1021/bi900574h 19583207 PMC2721637

[B23] Leimkühler S. Iobbi-Nivol C. (2016). Bacterial molybdoenzymes: old enzymes for new purposes. *FEMS Microbiol. Rev.* 40 1–18. 10.1093/femsre/fuv043 26468212

[B24] Liew F. Henstra A. M. Winzer K. Köpke M. Simpson S. D. Minton N. P. (2016). Insights into CO2 fixation pathway of *Clostridium autoethanogenum* by targeted mutagenesis. *mBio* 7:e00427-16. 10.1128/mBio.00427-16 27222467 PMC4895105

[B25] Lindahl P. A. (2002). The Ni-containing carbon monoxide dehydrogenase family: light at the end of the tunnel? *Biochemistry* 41 2097–2105. 10.1021/bi015932+ 11841199

[B26] Marreiros B. C. Calisto F. Castro P. J. Duarte A. M. Sena F. V. Silva A. F. (2016). Exploring membrane respiratory chains. *Biochim. Biophys. Acta* 1857 1039–1067. 10.1016/j.bbabio.2016.03.028 27044012

[B27] Martin W. F. Thauer R. K. (2017). Energy in ancient metabolism. *Cell* 168 953–955. 10.1016/j.cell.2017.02.032 28283068

[B28] Milton R. D. Ruth J. C. Deutzmann J. S. Spormann A. M. (2018). *Methanococcus maripaludis* employs three functional heterodisulfide reductase complexes for flavin-based electron bifurcation using hydrogen and formate. *Biochemistry* 57 4848–4857. 10.1021/acs.biochem.8b00662 30010323

[B29] Mock J. Wang S. Huang H. Kahnt J. Thauer R. K. (2014). Evidence for a hexaheteromeric methylenetetrahydrofolate reductase in *Moorella thermoacetica*. *J. Bacteriol.* 196 3303–3314. 10.1128/JB.01839-14 25002540 PMC4135698

[B30] Mock J. Zheng Y. Mueller A. P. Ly S. Tran L. Segovia S. (2015). Energy conservation associated with ethanol formation from H2 and CO2 in *Clostridium autoethanogenum* involving electron bifurcation. *J. Bacteriol.* 197 2965–2980. 10.1128/JB.00399-15 26148714 PMC4542177

[B31] Müller V. (2019). New horizons in acetogenic conversion of one-carbon substrates and biological hydrogen storage. *Trends Biotechnol.* 37 1344–1354. 10.1016/j.tibtech.2019.05.008 31257058

[B32] Peters J. W. Beratan D. N. Bothner B. Dyer R. B. Harwood C. S. Heiden Z. M. (2018). A new era for electron bifurcation. *Curr. Opin. Chem. Biol.* 47 32–38. 10.1016/j.cbpa.2018.07.026 30077080 PMC9113080

[B33] Ragsdale S. W. (2008). Enzymology of the Wood-Ljungdahl pathway of acetogenesis. *Ann. N. Y. Acad. Sci.* 1125 129–136. 10.1196/annals.1419.015 18378591 PMC3040112

[B34] Ragsdale S. W. Kumar M. (1996). Nickel-containing carbon monoxide dehydrogenase/acetyl-CoA synthase. *Chem. Rev.* 96 2515–2540. 10.1021/cr950058+ 11848835

[B35] Ragsdale S. W. Ljungdahl L. G. DerVartanian D. V. (1983). Isolation of carbon monoxide dehydrogenase from *Acetobacterium woodii* and comparison of its properties with those of the *Clostridium thermoaceticum* enzyme. *J. Bacteriol.* 155 1224–1237. 10.1128/jb.155.3.1224-1237.1983 6309745 PMC217820

[B36] Ragsdale S. W. Pierce E. (2008). Acetogenesis and the Wood-Ljungdahl pathway of CO2 fixation. *Biochim. Biophys. Acta* 1784 1873–1898. 10.1016/j.bbapap.2008.08.012 18801467 PMC2646786

[B37] Schäfer G. Engelhard M. Müller V. (1999). Bioenergetics of the archaea. *Microbiol. Mol. Biol. Rev.* 63 570–620. 10477309 10.1128/mmbr.63.3.570-620.1999 PMC103747

[B38] Schuchmann K. Müller V. (2012). A bacterial electron-bifurcating hydrogenase. *J. Biol. Chem.* 287 31165–31171. 10.1074/jbc.M112.395038 22810230 PMC3438948

[B39] Schuchmann K. Müller V. (2013). Direct and reversible hydrogenation of CO2 to formate by a bacterial carbon dioxide reductase. *Science* 342 1382–1385. 10.1126/science.1244758 24337298

[B40] Schuchmann K. Müller V. (2014). Autotrophy at the thermodynamic limit of life: a model for energy conservation in acetogenic bacteria. *Nat. Rev. Microbiol.* 12 809–821. 10.1038/nrmicro3365 25383604

[B41] Sousa F. L. Thiergart T. Landan G. Nelson-Sathi S. Pereira I. A. Allen J. F. (2013). Early bioenergetic evolution. *Phil. Trans. R. Soc. B* 368:20130088. 10.1098/rstb.2013.0088 23754820 PMC3685469

[B42] Thauer R. K. (1972). CO2-reduction to formate by NADPH. The initial step in the total synthesis of acetate from CO2 in *Clostridium thermoaceticum*. *FEBS Lett.* 27 111–115. 10.1016/0014-5793(72)80421-6 11946819

[B43] Thauer R. K. (2012). The Wolfe cycle comes full circle. *Proc. Natl. Acad. Sci. U.S.A.* 109 15084–15085. 10.1073/pnas.1213193109 22955879 PMC3458314

[B44] Thauer R. K. Kaster A. K. Seedorf H. Buckel W. Hedderich R. (2008). Methanogenic archaea: ecologically relevant differences in energy conservation. *Nat. Rev. Microbiol.* 6 579–591. 10.1038/nrmicro1931 18587410

[B45] Wagner T. Ermler U. Shima S. (2016). The methanogenic CO2 reducing-and-fixing enzyme is bifunctional and contains 46 [4Fe-4S] clusters. *Science* 354 114–117. 10.1126/science.aaf9284 27846502

[B46] Wagner T. Ermler U. Shima S. (2018). “Tungsten-containing formylmethanofuran dehydrogenase,” in *Encyclopedia of Inorganic and Bioinorganic Chemistry (online)*, ed. Messerschmidt A. (Hoboken: John Wiley and Sons, Inc).

[B47] Wagner T. Koch J. Ermler U. Shima S. (2017). Methanogenic heterodisulfide reductase (HdrABC-MvhAGD) uses two noncubane [4Fe-4S] clusters for reduction. *Science* 357 699–703. 10.1126/science.aan0425 28818947

[B48] Wang S. Huang H. Kahnt J. Mueller A. P. Köpke M. Thauer R. K. (2013). NADP-specific electron-bifurcating [FeFe]-hydrogenase in a functional complex with formate dehydrogenase in *Clostridium autoethanogenum* grown on CO. *J. Bacteriol.* 195 4373–4386. 10.1128/JB.00678-13 23893107 PMC3807470

[B49] Yamamoto I. Saiki T. Liu S. M. Ljungdahl L. G. (1983). Purification and properties of NADP-dependent formate dehydrogenase from *Clostridium thermoaceticum*, a tungsten-selenium-iron protein. *J. Biol. Chem.* 258 1826–1832. 6822536

